# Critically ill, tubular injury, delayed early recovery: characteristics of acute kidney disease with renal oxalosis

**DOI:** 10.1080/0886022X.2021.1885443

**Published:** 2021-03-04

**Authors:** Jing Zhou, Xiaojuan Yu, Tao Su, Suxia Wang, Li Yang

**Affiliations:** a Renal Division, Department of Medicine, Peking University First Hospital, Peking University Institute of Nephrology, Beijing, China; b Renal Pathology Center, Institute of Nephrology, Beijing, China; c Renal Division, Department of Medicine, Kailuan General Hospital, Tangshan, China; d Laboratory of Electron Microscopy, Pathological Center, Peking University First Hospital, Beijing, China

**Keywords:** Renal oxalosis, acute kidney disease, acute kidney injury, renal tubular injury, drug-induced

## Abstract

**Objects:**

This study aimed to analyze the clinicopathological features of acute kidney disease (AKD) with renal oxalosis.

**Methods:**

Data for biopsy-proven AKD with oxalosis diagnosed from Jan 2011 to Oct 2018 was collected. The underlying diseases, dietary habits, clinical and pathological characteristics of newly emerging kidney disease were analyzed. The long-term renal prognosis was observed.

**Results:**

A total of 23 patients were included, comprised of 18 men and 5 women with a mean age of 51.6 ± 15.9 years. The peak Scr was 669.9 ± 299.8 μmol/L, and 95.7% of patients had stage 3 acute kidney injury (AKI). Drug-induced tubulointerstitial nephritis (TIN) was the most common cause (65.2%) of AKD, followed by severe nephrotic syndrome (17.4%). All patients had pathological changes indicating TIN, and 11 patients were complicated with the newly emerging glomerular disease (GD). The risk of oxalosis caused by increased enterogenous oxalate absorption accounted for only 26.1%, and others came from new kidney diseases. The majority (75%) of abundant (medium to severe) oxalosis occurred in patients without GD. There were no significant differences in the score for tubular injury (T-IS) and interstitial inflammation with different severities of oxalosis. Rate of Scr decrease (ΔScr%) at 2 weeks was negatively correlated with the severity of oxalosis (*R* = −0.542, *p* = 0.037), score for T-IS (*R* = −0.553, *p* = 0.033), and age (*R* = −0.736, *p* = 0.002). The decrease in Scr at 4 weeks was correlated with T-IS (*R* = −0.433), but had no correlation with oxalosis.

**Conclusions:**

The present findings revealed that 95.7% of AKD with secondary renal oxalosis occurred in critically ill patients. AKD from tubular injury was the prominent cause. Severe oxalosis contributed to delayed early recovery of AKD.

## Introduction

Renal oxalosis is a disease in which large numbers of oxalate crystals are deposited in the renal tubules and renal interstitium, resulting in structural and functional disorders of the kidney. It is also known as oxalate nephropathy. Oxalate is an intermediate product of energy metabolism in the liver. Classical primary hyperoxaluria (PH) is a genetic disease in which abnormal glyoxylate metabolic enzymes in the liver lead to excessive accumulation of endogenous oxalate. The clinical manifestations are chronic damage to renal function and recurrent calcium oxalate kidney stones. Oxalate is mainly excreted by glomerular free filtration in the urine and is a representative small-molecule uremic toxin. Serum oxalate levels in patients with chronic kidney disease (CKD) gradually increase with the deterioration of renal function. High oxalate diet intake and intestinal fat malabsorption increase absorption of enterogenous oxalate, which can aggravate hyperoxaluria, leading to crystallization and inflammatory damage as adverse factors that promote CKD progression and increase the risk of cardiovascular disease [1–3].

Renal oxalosis can lead to acute tubular injury, which is frequently observed in patients after kidney transplantation and results in delayed recovery of renal allograft function. In clinical practice, oxalosis can also be found in patients with acute kidney injury (AKI) without kidney transplantation, but its mechanism and participation in the process of acute injury remain unclear. However, researchers still tend to focus on case reports for this disease [4–7], which lack summaries of relevant findings. The present study aimed to summarize the clinicopathological features of patients with acute kidney disease (AKD) and renal oxalosis who were diagnosed and followed up in our department, to further understand the characteristics of the disease and their effects on prognosis.

## Materials and methods

### Subjects

From January 2011 to October 2018, 23 cases of AKD diagnosed according to the KDIGO criteria with renal oxalosis confirmed by kidney biopsy were collected from Peking University First Hospital. The inclusion criteria were: (1) clinical diagnosis of AKD according to the KDIGO criteria [8,9]; and (2) presence of transparent or yellowish oxalate crystals in renal tissues detected by light microscopy, with birefringence under polarized light on HE staining. Patients who meet the inclusion criteria but were finally identified chronic kidney injury by renal pathological findings would be excluded from the study.

### Analysis of clinical, laboratory, and imaging data

Data on patient age, sex, underlying diseases (diabetes, hypertension, hyperuricemia, digestive system diseases, urinary system diseases, malignant tumors, other diseases), eating habits, and recent use of drugs (antibiotics, antiviral drugs, non-steroidal antipyretic analgesics) were collected. Vital signs, urine volume, medications for kidney disease during hospitalization, and renal prognosis were recorded. The evaluation criteria for renal outcomes were Scr levels and percentages of Scr decreases at 2 weeks to 6 months after the peak Scr was observed.

Routine urine examinations, microscopic examinations of urinary sediment, and analyses of urinary protein components were performed in all patients, including urine specific gravity, pH value, glucose, N-acetyl-β-D-glucosaminidase (NAG), and α1-microglobulin (α1-MG) before kidney biopsy. Data on 24-h urinary creatinine, uric acid, and electrolytes were collected, and the solute clearance rate and excretion fraction were calculated together with the results of blood tests. All patients were examined by ultrasonography to determine whether they were complicated with kidney stones.

### Evaluation of renal pathological features

Renal biopsy specimens were examined by fluorescence, light microscopy, and electron microscopy. The pathological diagnosis and renal tubulointerstitial injury score (TiIS) were made by two experienced renal pathologists. The modified renal TiIS was determined in accordance with the Banff working group classification standards [10,11], using the severities of tubular epithelial cell injury, interstitial edema, interstitial cell infiltration, tubular atrophy, and interstitial fibrosis, that they were scored 0, 1, 2, 3, and 4. According to the presence or absence of tubular cell necrosis and tubulitis, they were scored 0 or 1. The renal tubulointerstitial acute injury score (A-TiIS) was defined as the sum of the tubular injury score (T-IS) and interstitial acute inflammation score. The renal tubulointerstitial chronic injury score (C-TiIS) was defined as the sum of the renal tubular atrophy score, renal interstitial fibrosis score, and chronic inflammation score, which followed the same scoring rules as A-TiIS.

Oxalate crystal deposition was defined as transparent or light yellow clusters or radial crystals observed by optical microscopy that exhibited birefringence under HE staining with polarized light. Based on the total numbers of oxalate crystals deposited in 10 visual fields at 200× magnification, oxalosis was divided into three types: mild, 0–5; medium, 5–10; severe, >10.

### Statistical analysis

SPSS software version 20.0 (IBM Corp., Armonk, NY) was used for all statistical analyses to determine the severity of renal oxalosis and to compare and analyze the related influencing factors. Continuous variables with a normal distribution were expressed as mean ± standard deviation and compared between groups by a *t*-test, while continuous variables with a non-normal distribution were expressed as quartiles and compared between groups by a nonparametric test. Classified variables were expressed by a number of cases (percentage), with unordered variables compared by the Chi-square test and ordered variables compared by the Wilcoxon rank-sum test. Values of *p* < 0.05 were considered to indicate statistical significance (bilateral test).

## Results

### General conditions and clinicopathological features of AKD patients with renal oxalosis

A total of 23 patients with AKD and renal oxalosis were included in the study. These patients comprised 18 men (78.3%) and 5 women (21.7%) with a mean age of 51.57 ± 15.89 years. Among them, 22 patients (95.7%) met the KDIGO criteria for AKI and reached stage 3, 5 patients had the nephrotic syndrome, and 6 patients had nephritis syndrome.

The renal biopsy examinations showed that 11 patients had the glomerular disease (GD) complicated with the acute tubulointerstitial disease (tubulointerstitial nephritis [TIN]), including minimal change nephropathy, membranous nephropathy, lupus nephritis, IgA nephropathy, malignant hypertensive thrombotic microangiopathy, anti-glomerular basement membrane disease, post-infection glomerulonephritis, and diabetic nephropathy. The other 12 patients were diagnosed with the acute tubulointerstitial disease without GD. As shown in Table 1, combination with further etiological analyses by pathology revealed that the pathogenic factors leading to AKD were drugs induced TIN (15 cases, 65.2%), severe nephrotic syndrome (4 cases, 17.4%), severe glomerulonephritis (3 cases, 13.0%), infection (2 cases, 8.7%). The types of pathogenic drugs involved included non-steroidal antipyretic analgesics (NSAIDs, 53.3%), antibiotics (5 cases, 33.3%), antiviral drugs, platinum chemotherapeutic drugs, and traditional Chinese herbs. No cases with kidney transplantation or PH were observed. The prominent risk factors contributing to renal oxalosis as listed in Table 2 were sudden emergence of new kidney diseases, while increased enterogenous oxalate absorption occurred in only 6 cases (26.1%), including 3 patients with high dietary oxalate intake, 2 patients with refractory diarrhea and 1 patient after total gastrectomy.

**Table 1.** Pathogenic factors leading to AKD.

	AKD, oxalosis, with GD (*n* = 11)	AKD, oxalosis, without GD (*n* = 12)
Glomerulonephritis	3 (27.3%)	0
Nephrotic syndrome	4 (36.4%)	0
Increased oxalate-rich diet intake	1 (9.1%)	2 (16.7%)
Diarrhea	1 (9.1%)	2 (16.7%)
Infection	0	2 (16.7%)
Causative drugs	6 (54.5%)	9 (75.0%)
Antibiotics	2 (18.2%)	3 (33.3%)
NSAIDs	4 (36.4%)	4 (66.7%)
Others	0	4 (66.7%)

*Note*. NSAIDs. non-steroidal antipyretic analgesics. There may be one or more pathogenic factors for AKD.

**Table 2.** Risk factors contributing to renal oxalosis.

Risk factors	Cases
One identified risk factor	
New AKD	17
Increased enterogenousoxalate absorption due to fat malabsorption by diarrhea	1
Increased dietary intake through excessive consumption	1
More than two identified risk factors	
New AKD + increased enterogenousoxalate absorption	2
New AKD + increased dietary intake	2

*Note*. AKD: acute kidney disease.

Underlying diseases included one case with a history of kidney stones and 2 cases (8.7%) diagnosed with kidney stones by ultrasonography after hospital admission. There were 11 patients with diabetes (47.8%) and 17 patients with hypertension (73.9%). The mean body mass index (BMI) was 24.94 ± 4.42 kg/m^2^.

### Laboratory parameters characteristics of AKD patients with renal oxalosis

The mean peak Scr level in the patients was 669.9 ± 299.8 μmol/L. Twenty-two patients met the KDIGO criteria for AKI and reached stage 3, with 10 patients (45.5%) receiving hemodialysis at the time of disease onset. As shown in Table 3, urine volume was significantly decreased in patients with GD, with a mean urine output of 882.7 mL, and 63.6% showing <1000 mL on the second day of admission. Patients without GD, had both higher serum (464.4 ± 90.4 μmol/L vs. 337.6 ± 117.9 μmol/L, *p* = 0.01) and urinary (2793.3 ± 878.0 μmol vs. 1791.50 ± 1141.8 μmol, *p* = 0.312) uric acid concentrations, but the calculated uric acid excretion fraction was similar to that in patients with GD (21 vs. 22%, *p* > 0.05). The change in blood electrolytes was slight, and there were no significant differences in serum phosphorus (1.47 ± 0.44 mmol/L), corrected serum calcium (2.38 ± 0.14 mmol/L), sodium (138.31 ± 3.14 mmol/L), chlorine (105.02 ± 4.86 mmol/L), and potassium (4.14 ± 0.77 mmol/L) among AKD patients with oxalosis.

**Table 3.** Physical and laboratory findings.

	Total AKD, oxalosis (*n* = 23)	AKD, oxalosis, with GD (*n* = 11)	AKD, oxalosis, w/o GD (*n* = 12)	*t/Z* value	*p-*Value
BMI (kg/m^2^)	24.94 ± 4.42	24.79 ± 5.19	25.07 ± 3.85	0.14	0.89
SBP, mmHg	138.9 ± 14.3	135.18 ± 13.0	142.25 ± 15.2	1.196	0.245
DBP, mmHg	82.5 ± 9.3	82.5 ± 11.2	82.5 ± 7.7	0.011	0.991
BNP, pg/nL	250.0 (24.0, 869.0) (*n* = 17)	451.0* (179.0, 2133.5) (*n* = 9)	53.5* (11.5, 338.5) (*n* = 8)	−2.117	0.034
UV (mL/d)	1503.6 ± 605.8	882.7 ± 664.0	1572.7 ± 925.0	2.01	0.058
UP g/24h	0.21 (0.06, 1.85) (*n* = 22)	2.49* (0.34, 19.81) (*n* = 10)	0.07* (0.02, 0.16)	−3.236	0.001
U-PH	6.07 ± 1.07	6.55 ± 1.15*	5.63 ± 0.80*	−2.243	0.036
U-NAG (U/L)	26.00 (10.50, 78.50) (*n* = 21)	78.50* (32.75, 181.00) (*n* = 10)	13.00* (8.00, 26.00) (*n* = 11)	−2.996	0.003
α1-MG (mg/L)	71.80 (20.10, 171.00)	137.50* (83.30, 195.75)	27.40* (14.71, 57.40)	−2.613	0.009
U-Glu (%)	5/23 (21.7%)	3/11 (27.3%)	2/12* (16.7%)	0.379	0.64
GHbA1c (%)	7.05 ± 1.22 (*n* = 12)	6.00 ± 0.26 (*n* = 3)	7.40 ± 1.21 (*n* = 9)	1.927	0.083
SCr peak (µmol/L)	669.86 ±299.82	749.22 ±289.22	597.12 ±302.76	−1.229	0.233
SCr when biopsy (µmol/L)	407.89 ±257.14	423.49 ±271.52	393.60 ±254.48	−0.273	0.788
SUa (µmol/L)	401.0 ± 121.3	337.6 ± 117.9*	464.4 ± 90.4*	2.831	0.01
FeUa	0.21 ± 0.14 (*n* = 10)	0.21 ± 0.18 (*n* = 4)	0.22 ± 0.13 (*n* = 6)	0.123	0.905
CCa (mL/min)	0.42 ± 0.19 (*n* = 9)	0.38 ± 0.27 (*n* = 3)	0.44 ± 0.15 (*n* = 6)	0.422	0.686
S-Pi (mmol/L)	1.47 ± 0.44	1.46 ± 0.55	1.47 ± 0.33	0.071	0.944
FePi	0.33 ± 0.14 (*n* = 9)	0.27 ± 0.22 (*n* = 3)	0.35 ± 0.11 (*n* = 6)	0.75	0.478
Na (mmol/L)	138.31 ± 3.14	137.73 ± 3.62	138.85 ± 2.67	0.849	0.405
UNa (mmol/L)	61.07 ± 22.94 (*n* = 9)	72.67 ± 22.84 (*n* = 6)	52.38 ± 20.09 (*n* = 8)	−1.766	0.103
FeNa	0.04 ± 0.04 (*n* = 11)	0.06 ± 0.06 (*n* = 4)	0.03 ± 0.03 (*n* = 7)	−0.841	0.422
SK (mmol/L)	4.14 ± 0.77	3.94 ± 0.64	4.31 ± 0.87	1.149	0.263
UK (mmol/L)	19.57 ± 9.00 (*n* = 14)	25.67 ± 9.67* (*n* = 6)	15.00 ± 5.29* (*n* = 8)	−2.657	0.021
UK (mmol/d)	37.34 ± 16.45 (*n* = 14)	44.77 ± 19.17 (*n* = 6)	31.76 ± 12.53 (*n* = 8)	−1.539	0.15
UK/UCREA	4.17 ± 2.36 (*n* = 12)	5.33 ± 3.01* (*n* = 5)	3.33 ± 1.48* (*n* = 7)	5.010	0.049

*Note*. BMI: body mass index; SBP: systolic blood pressure; DBP: diastolic blood pressure; BNP: atrial natriuretic peptide; UV: urine output volume; UP: urinary proteinuria; U-PH: urine PH; U-NAG: urinary NAG enzyme; α1-MG: α1-microglobulin; SCr: serum creatinine; CCa: clearance of calcium; UK/UCREA: urinary potassium creatinine ratio. *The difference is significant between AKD with oxalosis subgroups.

Urinalysis showed that the patients had varying degrees of proteinuria, with 43.5% exhibiting microscopic hematuria. Patients with GD had more urinary protein at 0.34–19.81 g/day (median: 2.49 g/day), significantly higher urinary NAG enzyme, urinary α1-MG levels (median: 78.5 U/L and 137.5 mg/L, respectively), and urinary microalbumin (mA)/α1-MG ratio of 20.7 (range: 1.78–43.41) than patients without GD. In contrast, patients without GD rarely had proteinuria (range: 0.02–0.16 g/day; median: 0.07 g/day), had slightly increased urinary NAG and α1-MG levels compared with the reference values (13 U/mg/L and 27.4 mg/L, respectively), and having a lower mA/α1-MG ratio of 0.58 g/day (range: 0.35–1.2 g/day) when compared with GD patients. The incidence of renal glycosuria was only 21.7%. Oxalate crystals were observed in the urine before the kidney biopsy in only 2 patients (8.7%).

### Pathological features of AKD patients with renal oxalosis

**Figure 1.**
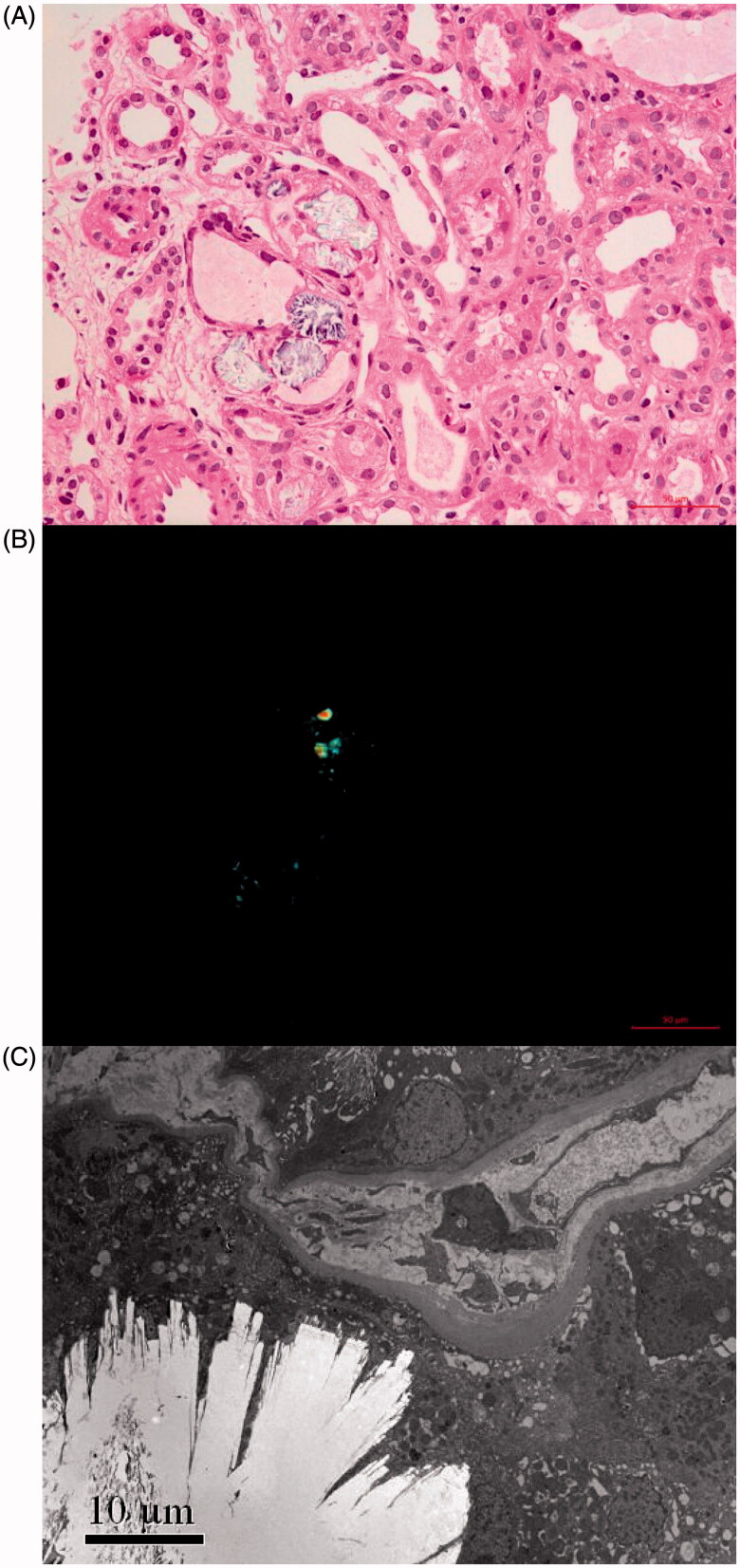
Renal oxalate crystal deposit. (A) Transparent or light brown crystal structures can be seen under light microscope (HE, ×400). (B) Birefringent crystals can be seen under polarized light microscope (HE, ×400). (C) Clusters of needlelike crystal structures under electron microscope (×3000).

In addition to the phenomenon of renal oxalosis, all 23 patients had the basic pathological features of acute TIN. According to the pathological scoring criteria used in this study, the scores for tubulointerstitial injury (Table 4) showed that regardless of the presence or absence of GD, the acute tubular injury was most prominent and interstitial lesions were mild. Pathologically, oxalate crystals showed mild to severe diffuse distribution in the renal tubule lumen (Figure 1) with surrounding secondary inflammatory reactions. The degree of oxalate crystal deposition was more diffuse in patients without GD, accounting for 50%, 8.3%, and 41.7% from mild to severe, while 75% were mild and only 8.3% were severe among patients with GD. Correlation analyses showed that the degree of oxalate crystal deposition was not correlated with urine volume, Scr level, total proteinuria, α1-MG level, renal glycosuria, or uric acid excretion rate, and was also not correlated with pathological scores for renal tubular injury. However when compared with mild oxalosis in patients without GD, the acute T-IS, I-IS, and Ti-IS of patients with abundant oxalate crystals deposition (medium and severe) were relatively higher: 4.21 ± 3.01 (vs.1.67 ± 1.15, *p* > 0.05), 2.00 ± 2.73 (vs. 0.0 ± 0.0, *p* > 0.05), 6.25 ± 4.59 (vs.1.67 ± 1.15, *p* = 0.029), respectively.

**Table 4.** Pathological scores.

	AKD, oxalosis, without GD	AKD, oxalosis, with GD
A-TiIS	5.0 ± 4.2	5.3 ± 3.4
T-IS	3.5 ± 2.7	4.2 ± 2.0
I-IS	1.5 ± 2.4	1.1 ± 2.1
C-TiIS	2.0 ± 1.8	1.9 ± 3.4
T-IS	0.67 ± 0.79	0.64 ± 1.21
I-IS	1.33 ± 1.16	1.27 ± 2.20

*Note*. AKD: acute kidney disease; GD: glomerulonephritis; A-TiIS: acute tubulointerstitial injury score; T-IS: tubular injury score; I-IS: interstitial injury score; C-TiIS: chronic tubulointerstitial injury score.

### Prognosis of AKD patients with oxalosis

Among the AKD patients with oxalosis in this study, 7 patients received prednisone combined with immunosuppressants for new GD, and 3 patients were administered 0.5 mg/kg initial dose of prednisone for tubulointerstitial disease. During follow-up until 6 months after diagnosis, 4 patients with severe glomerulopathy never recovered their renal function and maintained a state of dialysis, while the other 19 patients recovered. When compared with the peak Scr, ΔScr decreased by 50.2% at 2 weeks, 58.5% at 4 weeks, 62.5% at 8 weeks, and 67.9% at 24 weeks. Renal function in one patient with severe oxalosis caused by refractory diarrhea showed partial remission once under supportive treatment, but deteriorated again only 4 weeks later and ended at maintenance hemodialysis after 6 months. Statistical analyses revealed that the decrease rate of Scr after the peak (ΔScr%) was negatively correlated with the severity of oxalosis (*R* = −0.542), T-IS (*R* = −0.553), and age (*R* = −0.736). The decrease in Scr at 4 weeks was correlated with T-IS (*R* = −0.433) but had no correlation with the degree of oxalosis. This meant that the occurrence of oxalosis with AKD contributed to delayed early recovery of renal function.

## Discussion

This study describes characteristics of AKD with secondary renal oxalosis case series, which is clinically more common than PH, but a potentially underestimated disease. Oxalate crystals deposition in the kidney causes tubule obstruction and interstitial inflammation, leading to renal dysfunction [12,13]. As early as 1973, William R. Salyer and colleagues described oxalosis as a severe complication of chronic renal failure [14]. They observed renal and myocardial oxalosis affected 100%, 60% respectively among maintenance peritoneal dialysis patients. Meanwhile, Oxalate crystals were frequently present in autopsy patients with acute renal failure (90% with acute tubular necrosis), involving 56% of kidneys and 8% of the myocardium. In 2013, the Mayo Clinic reported a large group of 65 patients with renal oxalosis diagnosed by renal biopsies. The results showed that PH, kidney transplantation, and enterogenous hyperoxaluria or excessive food intake of oxalate synthesis precursors were the three major diseases, while only 6.2% were attributed to decreased renal function [12]. A meta-analysis of the causes for secondary renal oxalosis performed by researchers at Tufts University School of Medicine in the United States showed that 88% were secondary to fat malabsorption and 20% arose by excessive dietary oxalate consumption [15]. A recently published study reported oxalate nephropathy had a prevalence of 1% as the cause of native kidney disease. Disease origins from chronic pancreatitis and gastric bypass were the most common (48%) [13]. However, analysis of AKD with renal oxalosis in the present study showed a different etiological composition from that reported by Western researchers, where only 26.1% of patients having risk factors by enterogenous or excessive oxalate intake, but new AKD was regarded as the cause of oxalosis in these patients. Pathological findings revealed the tubular injury was the most prevalent feature having relevance to oxalosis. And the severity of tubular injury expressed by T-IS served as the key marker for predicting long-term prognosis. Drug-induced kidney injury came first in the pathogenic etiology of AKD, which deserved special attention.

The majority of oxalate in the blood is an endogenous product formed by glyoxylate metabolism in the liver. Oxalate precursors like vitamin C can also be converted into oxalate through liver metabolism. The proportion of enterogenous oxalate directly derived from oxalic acid-containing food absorbed through the gastrointestinal mucosa accounts for only 10–15% [16]. Small molecules of oxalate can be removed by glomerular free filtration, and excretion *via* the SLC26A transporter family on renal proximal tubular epithelial cells and intestinal mucosal epithelial cells is the active pathway for oxalate clearance from the body [17,18]. In disease states such as kidney failure, diabetes, and obesity, when oxalate cannot be fully excreted from the body through the urine and intestines, the concentration of oxalate in the blood will further increase, leading to hyperoxaluria [19]. In many subsequent studies, serum oxalate levels were directly measured. It was found that the serum oxalate level (median: 35 μmol/L) in patients with CKD was more than 3 times higher than the normal range [20]. Thus, severely impaired renal function is one of the most important reasons for secondary hyperoxalemia, hyperoxaluria, and increased risk of systemic tissue oxalosis. However, it is obvious that the elevation of serum oxalate in patients with PH and enterogenous hyperoxaluria (EH) is much higher than that with renal failure. It has been reported that when eGFR was 33 mL/min/1.73m^2^, plasma oxalate concentration were respectively 9.3, 4.1, 2.4 times higher than normal (PH > EH > CKD) [21]. Theoretically, urinary oxalate would be subsequently decreased as GFR declined. Whereas in this study, we noticed that in patients with AKD from severe GD, oxalosis was significantly mild. Furthermore, severe oxalosis mainly occurred in TIN relating to AKD. Their degree of diffuse oxalate crystal deposition was not correlated with Scr, but had a close relationship to pathological acute Ti-IS. We, therefore, speculate that urinary oxalate saturation resulting from renal dysfunction is not the only risk factor involved to oxalosis, that tubular injury itself might play a potential role in promoting crystallization. Previous research demonstrated that in an ethylene glycol-induced hyperoxaluria animal model, ischemia-induced renal tubular injury accelerated oxalate crystals deposition and aggravated the injury [22]. We notice that the transporter SLC26A6 which mediates active oxalate excretion is only expressed on the luminal surface of the S_3_ segment susceptible to ischemia. SLC26A6−/−knockout mice showed a prominent increase in blood and urinary oxalate levels, supporting the notion that renal tubular damage in S_3_ might induce subsequent hyperoxalemia and hyperoxaluria. SLC26A6 down-regulation yet weakens the inhibitory effect on the activity of the coupled citrate transporter NaDC-1, contributing to hypocitraturia that renders oxalate more likely to form crystals in urine [23].

In this study, we reported an average BMI of 24.94 ± 4.42 kg/m^2^, with a 47.8% of diabetes in AKD patients with renal oxalosis. A previous study found that patients with obesity and diabetes were often complicated with hyperoxaluria [24–26]. Animal experiments revealed that SLC26A6 activity in the jejunal epithelium of obese rats was significantly decreased, leading to a decrease in oxalate excretion through the intestine, which may also promote the occurrence of hyperoxaluria. Glyoxylate, the precursor of oxalate, was also identified as a metabolic marker closely related to the occurrence of diabetes in recent studies. The above evidence suggests metabolic syndrome could give rise to a possible higher risk of oxalosis in AKD patients.

Oxalate combines with calcium to form calcium oxalate crystals that are deposited in the renal parenchyma, phagocytosed by chemotactic macrophages, activate Nod-like receptors such as Nod-like receptor protein-3 (NLRP3), promote the secretion of inflammatory mediators like interleukin (IL)-1β and IL-8, trigger tissue inflammation and oxidative stress responses [27], and aggravate renal function damage. Analysis by Lumlertgul et al. [15] and Buysschaert et al. [13] pointed out the poor prognosis of renal oxalosis, with more than half of the patients needing to rely on dialysis. Increasing data from PH, EH, and CKD cohort support the contribution of plasma and urine oxalate to CKD progression [21,28]. For recipients after kidney transplantation, the high level of plasma oxalate was identified as the reason for the delayed graft function [20]. Based on the follow-up data in the present study, in addition to severe glomerular damage that renders some critically ill patients unable to stop dialysis, oxalosis not only reflects renal tubular damage but also indicates partial irreversibility of tubular injury. Therefore, there is an urgent need to develop novel strategies that can interfere with oxalate crystal formation to achieve a better renal outcome. Our study is an initial case series analysis with limitations. The most important limitation lies in being not available to measure oxalate concentration, which first needed to be further developed.

The results of this study indicate that secondary renal oxalosis mainly occurred in critically ill patients with tubular injury relating to AKD, which is associated with poor early recovery of renal function. Strategies to effectively ameliorate hyperoxalemia and hyperoxaluria may be helpful to the restoration of kidney function. All these should be guided based on oxalate-monitoring system.
